# Structural vulnerability in EPCR suggests functional modulation

**DOI:** 10.1038/s41598-024-53160-7

**Published:** 2024-01-31

**Authors:** Elena Erausquin, Adela Rodríguez-Fernández, Luis Ángel Rodríguez-Lumbreras, Juan Fernández-Recio, María Gilda Dichiara-Rodríguez, Jacinto López-Sagaseta

**Affiliations:** 1 Unit of Protein Crystallography and Structural Immunology, Navarrabiomed, 31008 Navarra, Spain; 2 Public University of Navarra (UPNA), Pamplona, 31008 Navarra, Spain; 3 Navarra University Hospital, 31008 Navarra, Spain; 4 Instituto de Ciencias de La Vid y del Vino (ICVV), CSIC–UR–Gobierno de La Rioja, 26007 Logroño, Spain

**Keywords:** Structural biology, Thrombosis

## Abstract

The endothelial protein C receptor (EPCR) is a fundamental component of the vascular system in mammals due to its contribution in maintaining blood in a non-prothrombotic state, which is crucial for overall life development. It accomplishes this by enhancing the conversion of protein C (PC) into the anticoagulant activated protein C (APC), with this property being dependent on a known EPCR conformation that enables direct interaction with PC/APC. In this study, we report a previously unidentified conformation of EPCR whereby Tyr154, critical for PC/APC binding, shows a striking non-canonical configuration. This unconventional form is incompatible with PC/APC binding, and reveals, for the first time, a region of structural vulnerability and potential modulation in EPCR. The identification of this malleability enhances our understanding of this receptor, prompting inquiries into the interplay between its plasticity and function, as well as its significance within the broader framework of EPCR's biology, which extends to immune conditions.

EPCR binds to protein C (PC) to accelerate the generation of its enzymatically active and anticoagulant form (activated PC, APC) to prevent excessive levels of thrombin in the bloodstream^1^. In humans, mutations in EPCR and anti-EPCR autoantibodies are associated with prothrombotic clinical outcomes^2^ and fetal demise^3,4^. At a molecular level, high-resolution crystal structures of the protein C Gla (vitamin K-dependent carboxylation/gamma-carboxyglutamic) domain bound to EPCR with its canonical conformation^5^, and alanine mutagenesis studies^6^, have pinpointed the specific amino acids in EPCR that contribute and are essential for protein C/APC binding. Among them, Tyr154 plays a pivotal role by establishing a robust network of interactions with the Gla domain, thus enabling binding of protein C and APC.

Crystallizations trials performed in our laboratory with the extracellular fraction of EPCR (hereafter EPCR) enabled the identification of an unusual conformation in a hinge region that connects two alpha helices present in the molecular structure of EPCR (Fig. 1). While the overall structure of the receptor adopts the canonical shape of EPCR^5,7^, i.e., a CD1/MHC-class I-like platform defined by two alpha helices on a β-sheet platform creating a tunnel for lipid binding, we noticed a striking conformational change in the α2 helix (Fig. 1A–D). Owing to the observed plasticity and structural vulnerability, we will refer to this specific region as the "*vul* region" (Fig. 1D). This novel conformation has a profound impact on the orientation of the Tyr154 side chain. Previous studies by Liaw and colleagues have demonstrated that Tyr154 side chain is essential for proper binding of protein C/APC to EPCR, as Tyr154 replacement with alanine results in an EPCR form unable to bind PC/APC^6^. This non-canonical rotamer of Tyr154 is the result of an alternative structural arrangement of a short region with undefined secondary structure that switches the direction of the α_2–1_ helix (Fig. 1A–C). This hinge-like motif can be seen as a structural piece that breaks the α2 helix into two independent helical rigid bodies, α_2–1_ and α_2–2_. This is a common feature also observed in MHC class I and II antigen-presenting molecules^8^.

**Figure 1:**
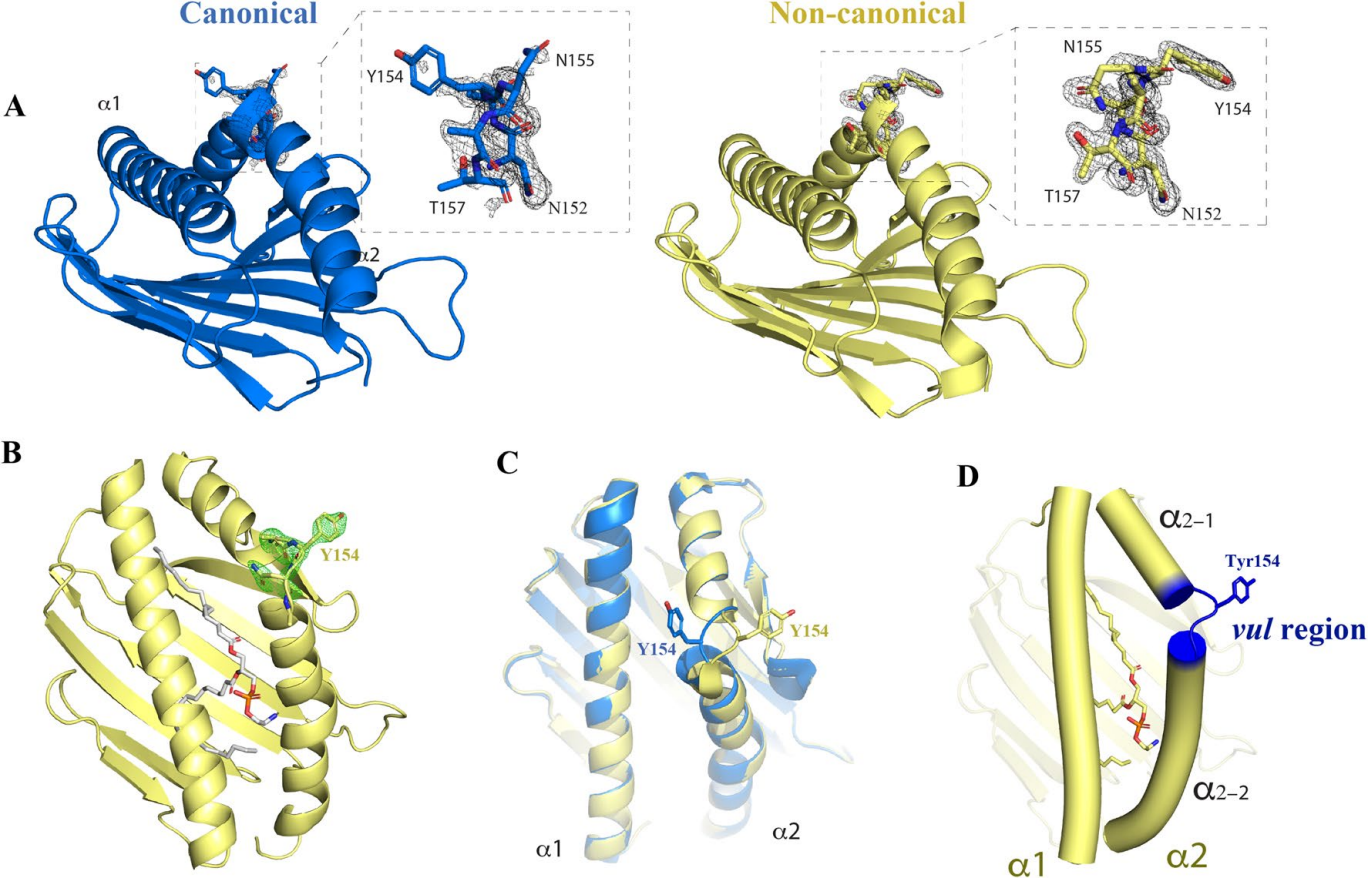
The non-canonical EPCR structure. (**A**) The canonical (left) and non-canonical EPCR structures are shown in blue and pale yellow colors, respectively. The residues in the α2 helix with severe folding transitions are highlighted. 2Fo-Fc electron density maps are shown as dark grey meshes. The lipid is not shown to facilitate the focus on the *vul* region **(B)** Residues Ala153-Tyr154-Asn155 were omitted from the protein coordinates, the structure refined and the Fo-Fc electron density map (green mesh) was calculated to show an unbiased signal of the *vul* region. **(C)** Superposition of the canonical and non-canonical structures with Tyr154 highlighted as sticks. The lipid is not shown. **(D)** Top view of EPCR with the alpha helices shown as cylinders and the structurally vulnerable region (*vul* region) highlighted in blue. The bound lipid is shown inside the EPCR cavity.

Analysis of the interaction between EPCR and the protein C Gla domain shows how Tyr154 establishes numerous Van der Waals contacts with protein C Gla backbone and Asn2 and Phe4 side chains (Fig. 2A). A hydrogen-bond with the γ-carboxyglutamic acid 7 (Gla7) further contributes to the overall network of interactions mediated by Tyr154. In this novel structure, Tyr154 shows a profound structural transition and alters the location of its side chain in a manner such that is completely away from the protein C binding site. The protein backbone at the *vul* region also presents a deep rearrangement, starting with a rotation of the Ala153 carbonyl by 90º and followed by severe conformational shifts that affect not only Tyr154 but Asn155, Arg156 and Thr157 peptide bonds angles and side chains (Figs. 1A, 2B). While unliganded EPCR structures show a highly flexible Arg156 side chain, as inferred from the lack of electron density signals in previously reported structures, the EPCR:protein C-Gla complex structure indicates that a precise and restricted configuration of Arg156 side chain is necessary for this interaction to occur. This plasticity in the Arg156 side chain suggests that EPCR exists as a heterogeneous population whereby only those EPCR molecules with Arg156 in a favorable conformation enable protein C/APC binding. Or alternatively, this flexibility favors Arg156 motion and protein C docking. This is consistent in our structure, where we do not observe an electron density signal for the Arg156 side chain. The receptor restores its canonical conformation from Arg158 onwards.

**Figure 2:**
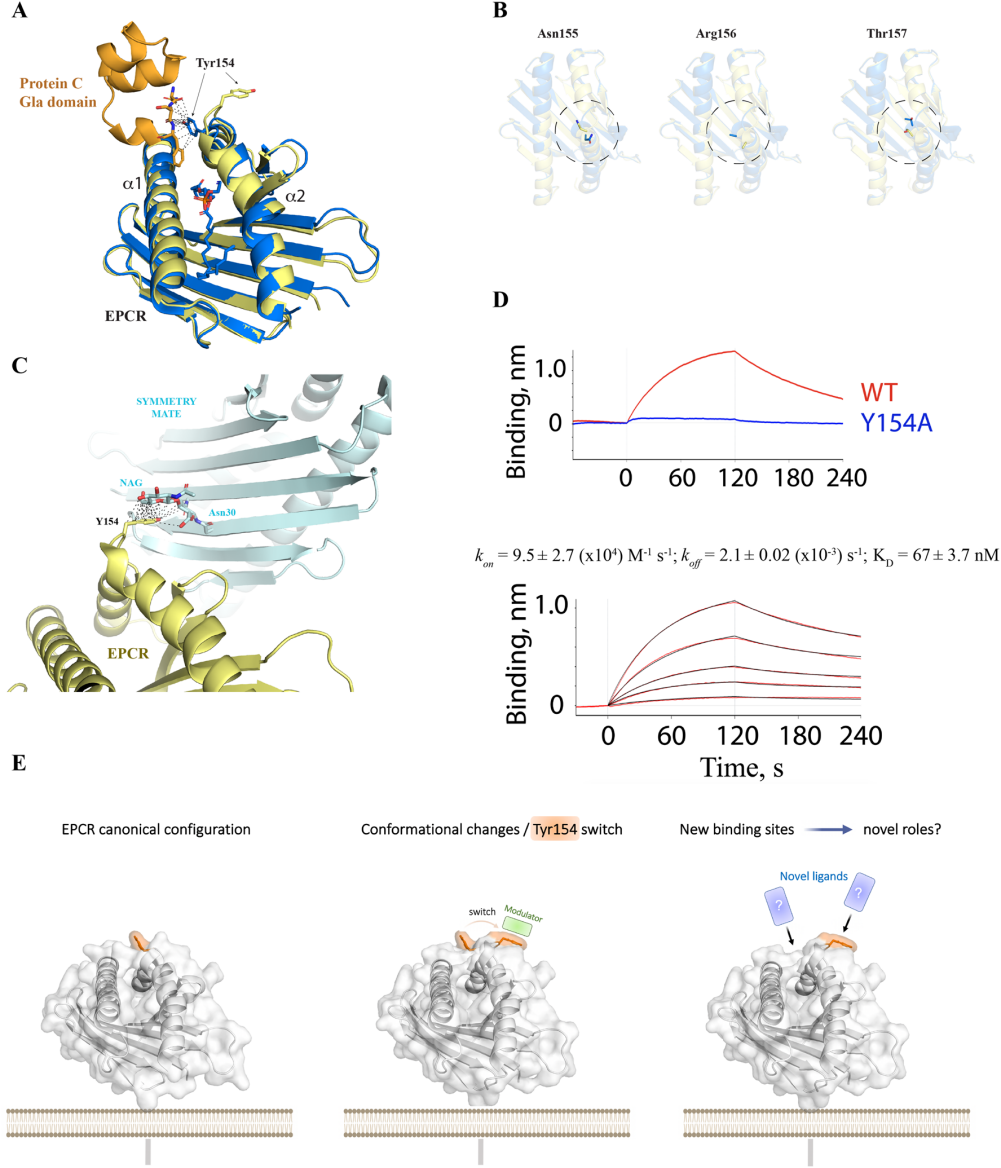
Impact of the non-canonical folding mode in protein C/APC binding. **(A)** Structure of the protein C Gla domain (in orange color) in complex with the canonical conformation of EPCR (PDB 1LQV). The contacts with the Gla domain established by EPCR Tyr154 are highlighted with grey dashed lines. Tyr154 residues in both the canonical and non-canonical EPCR structures are highlighted in sticks for comparison purposes and to better visualize the impact of the folding transition in protein C binding. The bound lipid can be observed inside the pocket as sticks. **(B)** Top views of Asn155, Arg156 and Thr157 in the *vul* region. Note that the side chain of Arg156 is omitted due to the lack of electron density **(C)** Intermolecular contacts (within 4 Å) established between Tyr154 side chain and the N-acetylglucosamine (NAG) present in a crystallographic symmetry mate molecule. **(D)** A direct and visual comparison of the binding profiles of wt EPCR and EPCR Y154A to 200 nM APC is shown in a biolayer interferometry sensorgram. The full binding kinetics with wt APC is displayed in the lower panel. Black and red lines indicate, respectively, the raw data and the fitting of the association and dissociation curves to a 1:1 binding model. **(E)** Shown is a graphical sketch depicting the diverse configurations that the *vul* region can acquire, as evidenced by X-ray studies. The presence of neighbouring molecules with affinity for EPCR could alter the canonical conformation, giving rise to novel binding sites and yet unknown biological properties.

To assess whether the conformational change observed in the crystal form (Fig. 2C) replicates the mode in which EPCR exists in solution, we performed molecular dynamic simulations (MDs) with EPCR as a monomer. In addition, we analyzed EPCR as a dimer using as initial conformation two neighbouring EPCR molecules as they appear in the novel crystal form (Fig. 2C), i.e.*, *with Tyr154 making contacts with the NAG sugar bound to Asn30 in the neighbouring EPCR (Fig. 2C).

The calculated trajectories denote that the canonical configuration is the dominant, yet not exclusive, EPCR form as a monomer (Supplementary Fig. 2A–C, Supplementary Movies 1–2). Remarkably, the presence of a neighbouring molecule, like N-acetylglucosamine in the crystallographic lattice (Fig. 2C, Supplementary Fig. 3), stabilizes the non-canonical conformation in the MDs (Supplementary Fig. 2D–E, Supplementary Fig. 4, Supplementary Movies 3–4), thus supporting the experimental observation.

To confirm the relevance of Tyr154 in protein C binding, we replaced it with alanine and monitored in real time the binding of EPCR Y154A to APC via biolayer interferometry. As expected, and confirming previous findings by other groups^6,9^, replacement of EPCR Tyr154 with alanine has a profound impact in APC binding (Fig. 2D). Consequently, these results further confirm the relevance of Tyr154 side chain in the binding of EPCR to protein C/APC, and support the notion that this alternative state of EPCR is not compatible with an anticoagulant role of the receptor.

In a manner similar to the CD1 family of proteins, EPCR is also known for binding lipids in a central cavity. Consequently, the nature and arrangement of the lipid in the new structure are of interest. In the novel structure, the electron density suggests that the lipid bound is phosphatidylethanolamine, which is consistent with previous reports^5,7,10^. Concerning the position of the lipid, we did not observe any relevant discrepancies with respect to previously reported EPCR structures (Supplementary Fig. 5). The differences with respect to the position of the lipid head group are consistent with previous observations indicating that the lipid exhibits a degree of mobility within the cavity.

An additional question to explore is whether a potential glycosylation in Asn155 could play a role in the context of these findings. Like in the novel structure, the previous crystal structure of EPCR alone (PDB 1L8J) did not show evident signs of N-glycosylation at Asn155. Yet, as already described, the conformations of their respective regions are drastically different, which suggests that the conformational change observed in the novel structure is not modulated by N-glycosylations at Asn155. Rather, by an inherent structural plasticity of the *vul* region. Indeed, the *vul* region could be defined as a coiled region given the lack of a well-defined regular structure. It is well known that such random coils contribute to the overall flexibility and shape of proteins. However, EPCR had not yet been demonstrated experimentally to present such plasticity.

Conformational heterogeneity and structural motion are inherent features often found in proteins. For instance, G protein-coupled receptors (GPCRs) possess structural motility that results essential for their biological properties^11^, and silent and active states coexist in equilibrium^12^. Numerous crystallization studies have led to a deep understanding of GPCR molecular plasticity, which results in a wide spectrum of structural, silent and active, states. These varied conformations are unique and link GPCRs to diverse biological fates. Another example of conformational plasticity is NFAT or the nuclear factor of activated T cells. X-ray structural studies of this transcription factor have shown evidence for the coexistence of a heterogeneous population of fairly diverse conformations^13^.

In this study we have identified a novel conformation of EPCR that features a deep structural motion in Tyr154 and surrounding residues. This plasticity gives rise to a novel configuration in a highly relevant region of EPCR with regard to its implications in the anticoagulation system. The structural plasticity of the *vul* region represents a site of vulnerability that could be modulated by alternative binders. The question remains whether this novel folding arrangement represents a structural binding motif for other EPCR ligands, and which could determine relevant yet unknown roles of EPCR. In this line, recent works suggest EPCR-dependent T cell^14–16^ and antibody^17^ recruitment, which indicates that EPCR can interact with a broad variety of protein molecules. Collectively, and in view of the results presented herein, the EPCR interactome and molecular plasticity is larger than originally expected, and further research is needed to address the molecular interaction potential of this receptor. In conclusion, our structural studies reveal a highly malleable region in EPCR whose conformation may dictate EPCR properties in blood coagulation and recognition by immune receptors.

Our results demonstrate that the presence of a structurally flexible region in EPCR that can alter its configuration and consequently the receptor properties, e.g., accelerated generation of anticoagulant APC, in the presence of a surrounding binding partner (Fig. 2E). Notably, the region housing this conformation contains amino acids that are crucial for mediating significant protein–protein interactions, hinting at its potential functional significance.

Importantly, it is likely that the crystallographic packing played a significant role in the occurrence of this novel configuration. However, while this study does not claim the presence of naturally-occurring multiple conformations in EPCR, it provides evidences for the presence of a region of structural vulnerability that could shape EPCR's properties (Fig. 2E).

In conclusion, these findings expand our comprehension of EPCR's functional diversity and raise questions about the specific role and relevance of this conformation within the broader context of EPCR's biology, thus warranting further exploration and research into this intriguing protein.

## Materials and methods

### EPCR crystallization and structure determination

The extracellular region of human EPCR was produced, digested and purified as described^7^. The purified product was crystallized in 20% PEG3500, 0.2 M NaK tartrate. A native and complete diffraction dataset [Resolution 1.8 Å, overall completeness 99.89%, high-resolution shell CC(1/2) 0.665, high-resolution shell I/sigma 1.69)] was collected and processed^18,19^. The new structure was solved via molecular replacement^20^ and polished with multiple steps of refinement^21,22^ combined with protein building^23^.

### Protein–protein interaction studies

EPCR or EPCR_TYR154A_ with a C-terminal 12xHis tag were captured on the surface of a NiNTA pre-coated biosensors (Sartorius) and the binding profiles of increasing concentrations of APC (Invitrogen) monitored in a Blitz system (Sartorius). The kinetic rate constants were calculated using a 1:1 Langmuir binding model through the Blitz Pro software (version 1.2.1.5).

### Molecular dynamics setup and protocol

Molecular dynamics (MD) were performed on the canonical (PDB ID 1L8J) and non-canonical (proposed in this work) structures of EPCR. The analysis was also extended to the dimeric EPCR forms found in the crystallization lattice for both the canonical and non-canonical structures. The parametrization of the protein coordinates was performed by the LEAP module from AmberTools^24^ using ff99SB^25^ and the general AMBER force field (GAFF)^26^. The phosphatidylethanolamine (PTY) cofactor was generated with the AMBER module ANTECHAMBER^24^ and LEAP. PTY ideal coordinates were obtained from the PDBeChem database^27^. The hydrogen in the carboxyl group was removed to adequate the protonation state to physiologic conditions, setting the net charge to -1, using AM1-BCC^28^ as a charge model. In the case of the dimeric structures, the N-glycosylated residues (Ans19-2xNAG, Ans30-2xNAG, and Ans47-2xNAG, the latter only in the canonical dimeric structure) were parametrized using a similar approach to the PTY. A fast minimization with cartesian restraints was performed to remove severe clashes, followed by minimization with explicit solvation. Each receptor was embedded in a solvated system within a periodic truncated octahedron box, and 150 mM NaCl was added to the system. Lastly, a fast solvent minimization was performed with a restraint mask of waters and ions. Then equilibration was performed at constant volume, using a 12 Å non-bonding cutoff. The equilibration process started by running 40 ps with protein constraints (50 kcal/mol Å^2^, from 0 to 300 K) using Langevin dynamics (LD). The following 40 ps restraints were reduced from 50 to 25 kcal/mol Å^2^*,* and the next 40 ps from 25 to 10 kcal/mol Å^2^ at constant pressure. Finally, restraints were reduced to 5 kcal/mol Å^2^, including backbone atoms, followed by 20 ps with backbone restraints (1 kcal/mol·Å^*2*^) and 60 ps with no restraints using LD, at 300 K. After equilibration, 100 ns MD were performed at 300 K at constant pressure. The MD of the dimeric structures were extended to 300 ns, and two additional runs of 100 ns MD were performed.

### LowModeMD

The LowModeMD^29^ module (Molecular Operating Environment, version 2022.03) was applied to search the folding space around the *vul* region in EPCR. More specifically, the search focused on molecular motions based on dihedral rotations in the target region, which was confined to the Gln150-Glu160 site. The procedure applies molecular dynamics along with distortions in an initial conformation within a low-frequency vibrational setting, complemented with search for energy *minima* in the last stage. In order to focus the analysis in the region of interest and to provide residues in the vicinity with motility freedom, we fixed all atoms but the Q150-E160 region. Parameters were set to an energy minimization root mean square gradient constraint of 0.100 and an energy window of 100 kcal mol^-1^ within an AMBER10:EHT forcefield.

### Supplementary Information

Supplementary Information 1.

Supplementary Figure 1.

Supplementary Figure 2.

Supplementary Figure 3.

Supplementary Figure 4.

Supplementary Figure 5.

Supplementary Video 1.

Supplementary Video 2.

Supplementary Video 3.

Supplementary Video 4.

## Data Availability

The atomic coordinates and structure factors are deposited in the Protein Data Bank: https://www.rcsb.org/, PDB ID 7Q5D, Structure of EPCR in a non-canonical conformation.
