# Supplementary material for: Structural vulnerability in EPCR suggests functional modulation
Source: Sci Rep. 2024 Jan 31;14:2591. doi: 10.1038/s41598-024-53160-7 (PMC10830566; doi:10.1038/s41598-024-53160-7)
Supplement: Supplementary file 1 — Supplementary Information 1. [file 41598_2024_53160_MOESM1_ESM.pdf]

SUPPLEMENTARY MATERIALS

Supplementary Figure 1. Configuration of the bound lipid in the non-canonical EPCR structure. The canonical and non-canonical structures are shown superposed on the alpha carbons. The position and conformation of the bound lipids, shown as sticks, can be noticed inside the hydrophobic groove.

Supplementary Figure 2. Conformational analysis of Tyr154 in the MD trajectories. **(A)** RMSD of Tyr154 residue in the different conformations sampled by 100 ns MD starting from the canonical structure, with respect to the canonical structure (orange) or the non-canonical structure (blue). **(B)** RMSD of Tyr154 in the different conformations sampled by 100 ns MD starting from the non-canonical structure, concerning the canonical (orange) or the non-canonical structures (blue). **(C)** The plasticity of the *vul* region was analyzed as a means of MD simulations based on dihedral rotations in the target region (Gln150-Glu160) and using the canonical structure (PDB 1L8J). The different series of stable configurations obtained are displayed and grouped according to the values of their energy minima. **(D)** RMSD of Tyr154 residue along the MD trajectory starting from the canonical dimeric structure, with regard to the canonical structure (orange) or the non-canonical structure (blue). **(E)** RMSD of Tyr154 residue along the MD trajectory starting from the non-canonical dimeric structure, relative to the canonical structure (orange) or the non-canonical structure (blue).

Supplementary Figure 3. Comparison of the crystallographic contacts in the crystal lattices. EPCR symmetry-related molecules in the crystal lattices are shown as cartoons. For comparison purposes, Tyr154 and the sugar molecules are highlighted as sticks in blue color.

Supplementary Figure 4. Extended 300 ns MD trajectories with crystal lattice EPCR dimers. RMSD of Tyr154 in the different MD conformations starting from the canonical **(A)** or the non-canonical **(B)** dimeric forms, with respect to the equivalent atoms in the canonical (orange) or in the non-canonical (blue) structures. To facilitate comparison, only one of the subunits of each dimer conformation was superimposed and used for the RMSD calculations. Representative structures (in green) randomly selected from the last 20 ns of the MD trajectories starting from the canonical **(C)** or the non-canonical **(D)** dimeric forms, are shown in ribbon, with Tyr154 residue as ball & sticks representation. The initial structures for the canonical (orange) and non-canonical (blue) dimeric structures are shown for comparison. Molecular images created with ICM-Browser (www.molsoft.com).

Supplementary Figure 5. Stereo view of EPCR. The 2Fo-Fc electron density maps around the *vul* region are displayed in stereo view.

Supplementary Video 1. Molecular dynamics simulations, canonical EPCR in its monomeric form. Molecular dynamics (MD) trajectories were calculated as described in Material and Methods section. The video captures an image every 100 picoseconds at Full HD resolution (1920x1080 pixels), and are animated at 25 frames per second, *i.e.*, 1 movie second is equivalent to 2.5 MD nanoseconds).

Supplementary Video 2. Molecular dynamics simulations, non-canonical EPCR in its monomeric form. Molecular dynamics (MD) trajectories were calculated as described in Supplementary Video 1.

Supplementary Video 3. Molecular dynamics simulations, canonical EPCR in its dimeric crystallographic form. Molecular dynamics (MD) trajectories were calculated as described in Supplementary Video 1.

Supplementary Video 4. Molecular dynamics simulations, non-canonical EPCR in its dimeric crystallographic form. Molecular dynamics (MD) trajectories were calculated as described in Supplementary Video 1.

Supplementary Table 1. X-ray data collection, processing, and refinement statistics.

**Parameter**	**Non-canonical EPCR**
Resolution range (Å)	96.64 - 1.8 (1.84 - 1.8)
Space group	$P3_121$
Unit cell	70.47 70.47 96.635 90 90 120
Total reflections	159141 (15874)
Unique reflections	26276 (2580)
Multiplicity	6.1 (6.2)
Completeness (%)	99.89 (99.96)
Mean I/sigma(I)	23.51 (1.69)
Wilson B-factor	42.16
R-merge	0.03282 (0.936)
R-meas	0.03603 (1.024)
R-pim	0.01458 (0.4107)
CC1/2	0.999 (0.665)
Reflections used in refinement	26273 (2580)
Reflections used for R-free	1398 (125)
R-work	0.1847 (0.3226)
R-free	0.1997 (0.4046)
RMS (bonds)	0.013
RMS (angles)	1.19
Ramachandran favored (%)	96.49
Ramachandran allowed (%)	3.51
Ramachandran outliers (%)	0.00
Average B-factor	59.44

Statistics for the highest-resolution shell are shown in parentheses.
