# Supplementary figures and images for: Structural vulnerability in EPCR suggests functional modulation
Source: Sci Rep. 2024 Jan 31;14:2591. doi: 10.1038/s41598-024-53160-7 (PMC10830566; doi:10.1038/s41598-024-53160-7)

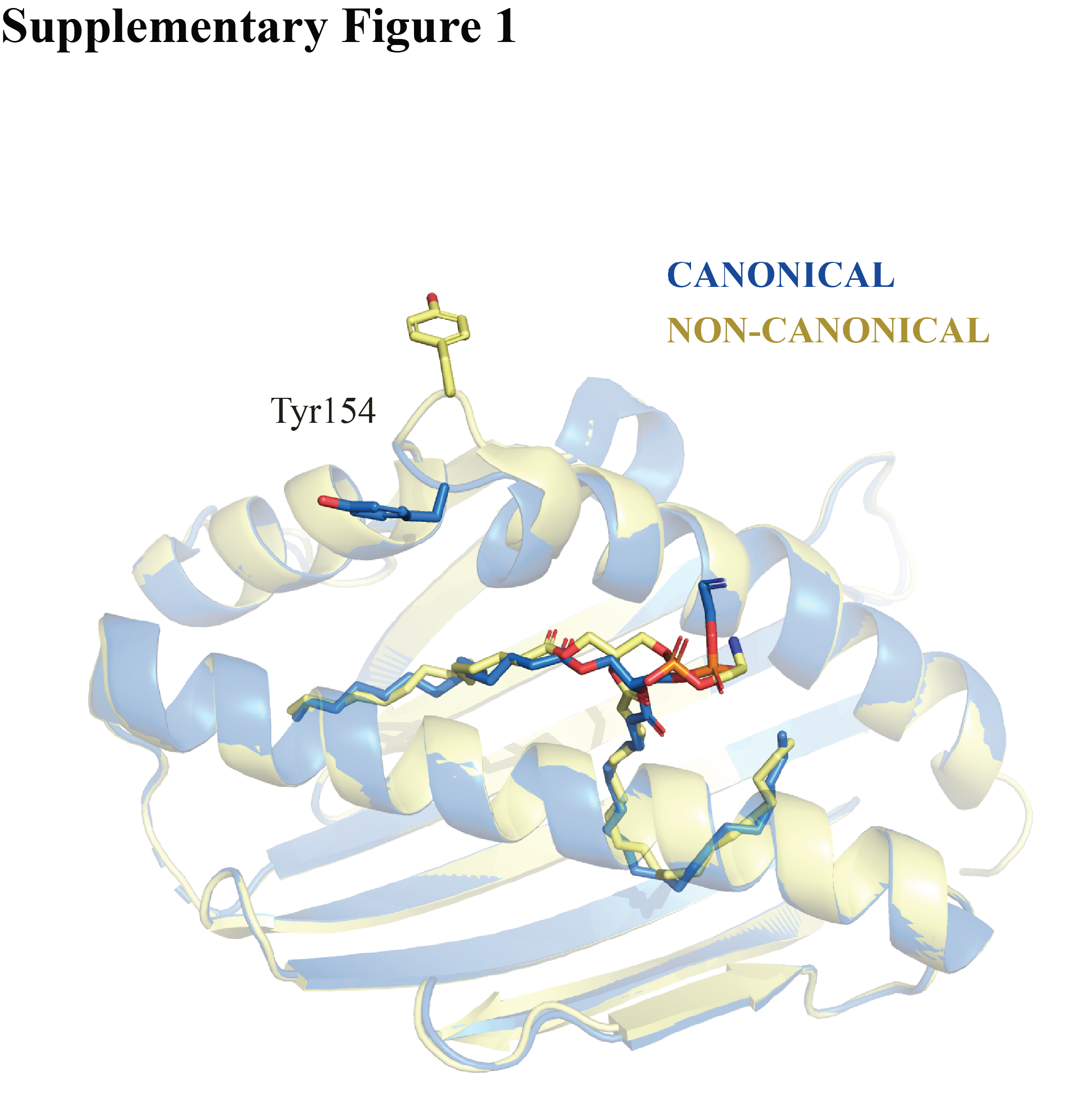

Supplement: Supplementary file 2 — Supplementary Figure 1. [file 41598_2024_53160_MOESM2_ESM.png]

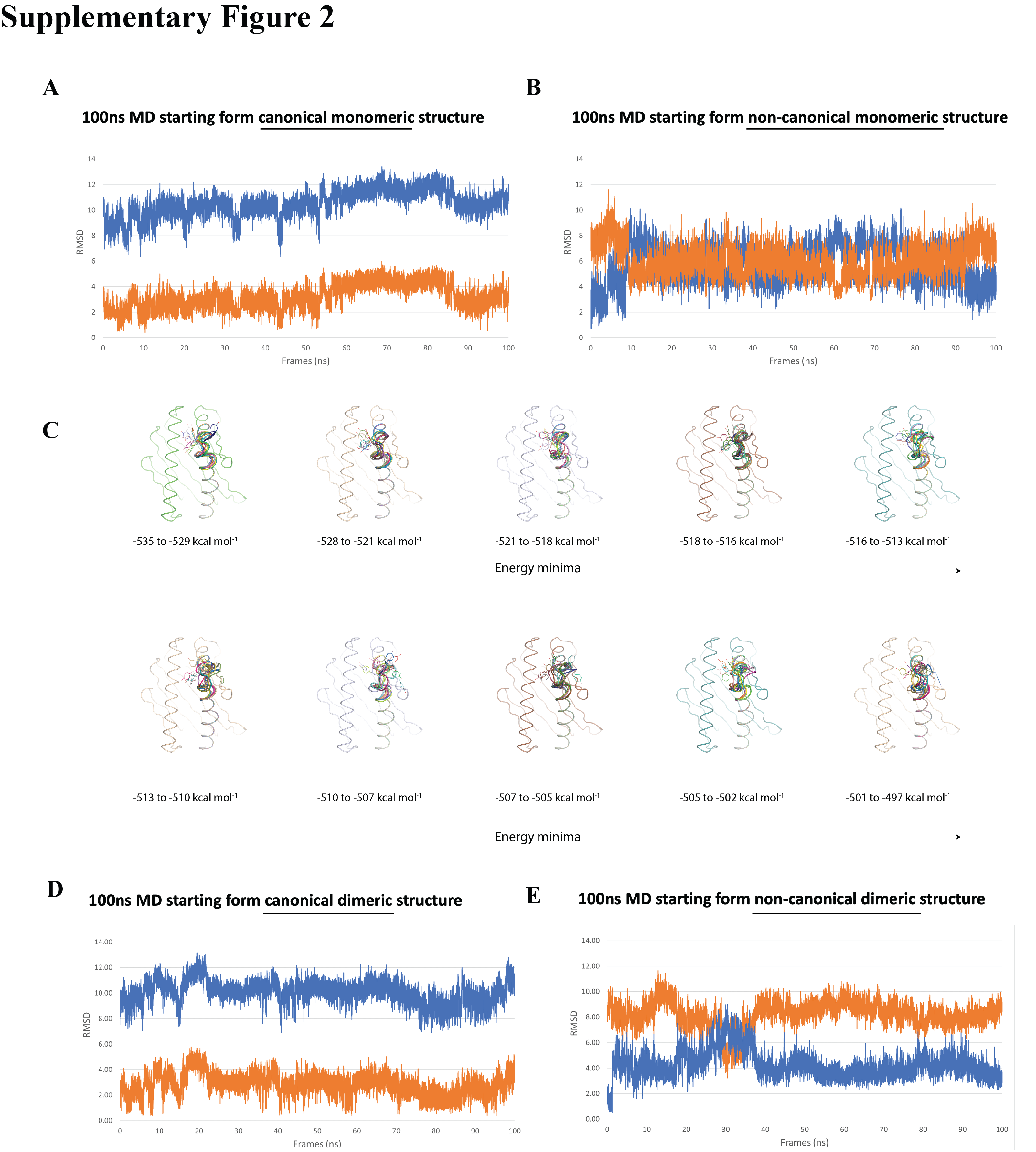

Supplement: Supplementary file 3 — Supplementary Figure 2. [file 41598_2024_53160_MOESM3_ESM.png]

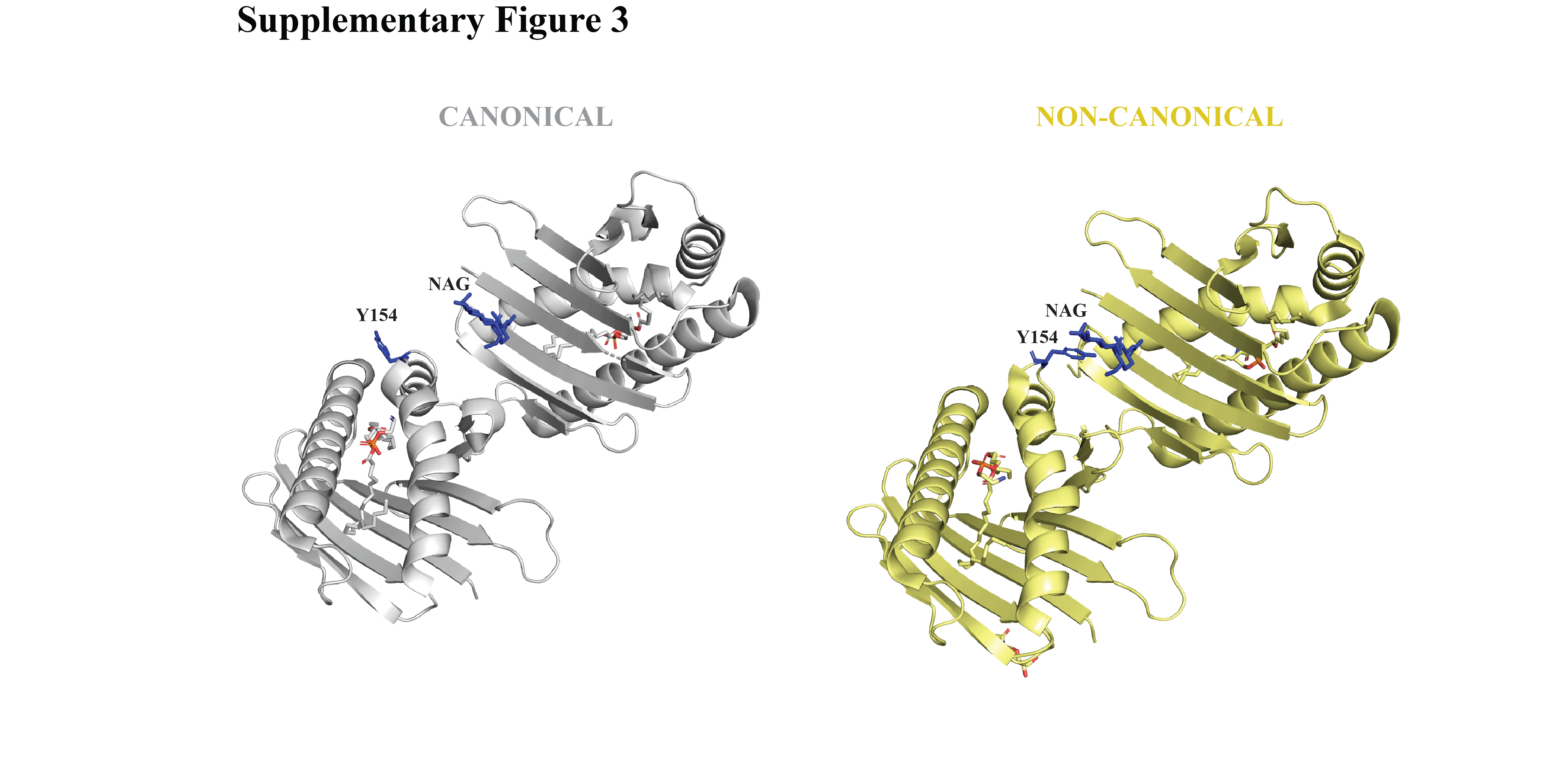

Supplement: Supplementary file 4 — Supplementary Figure 3. [file 41598_2024_53160_MOESM4_ESM.png]

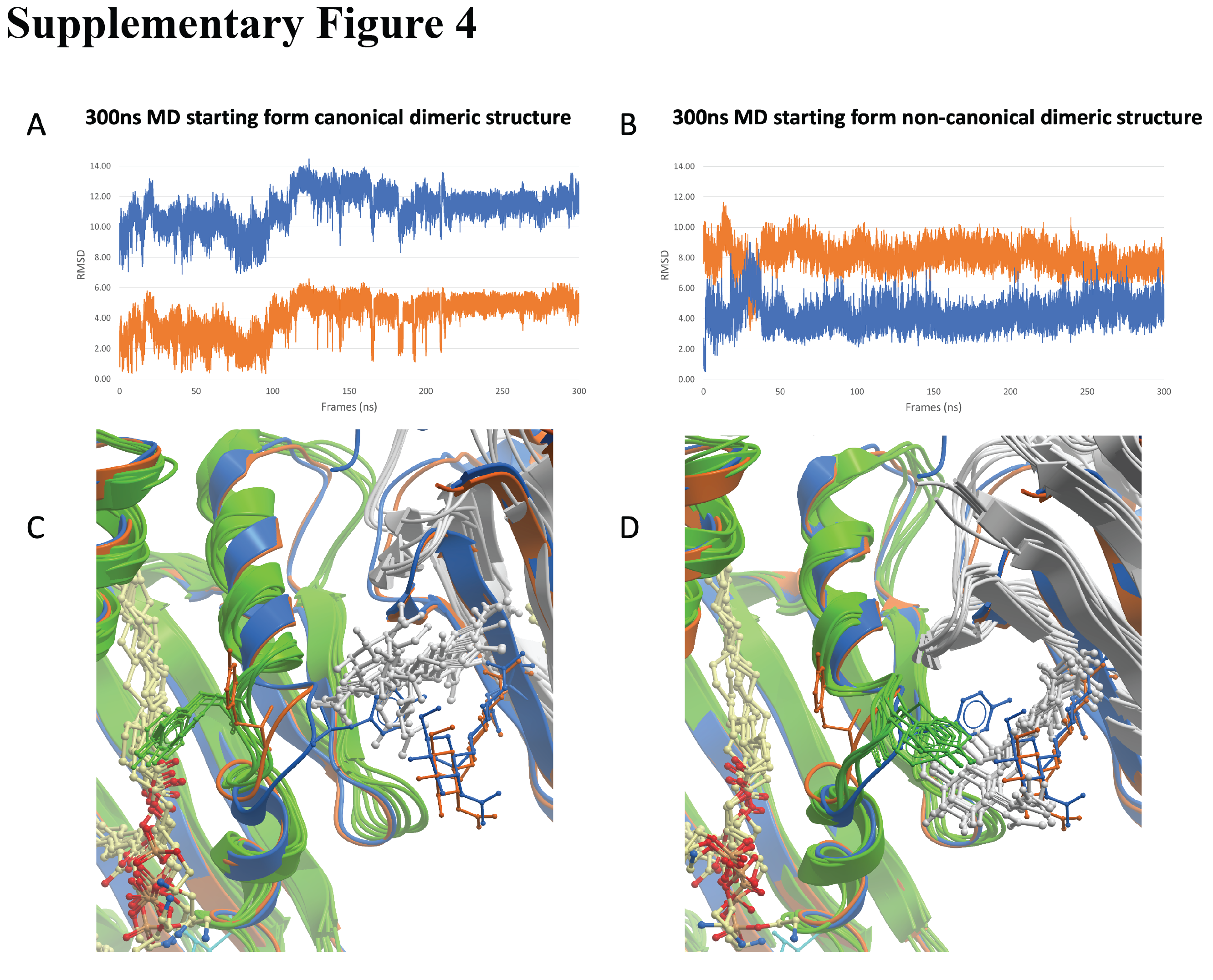

Supplement: Supplementary file 5 — Supplementary Figure 4. [file 41598_2024_53160_MOESM5_ESM.png]

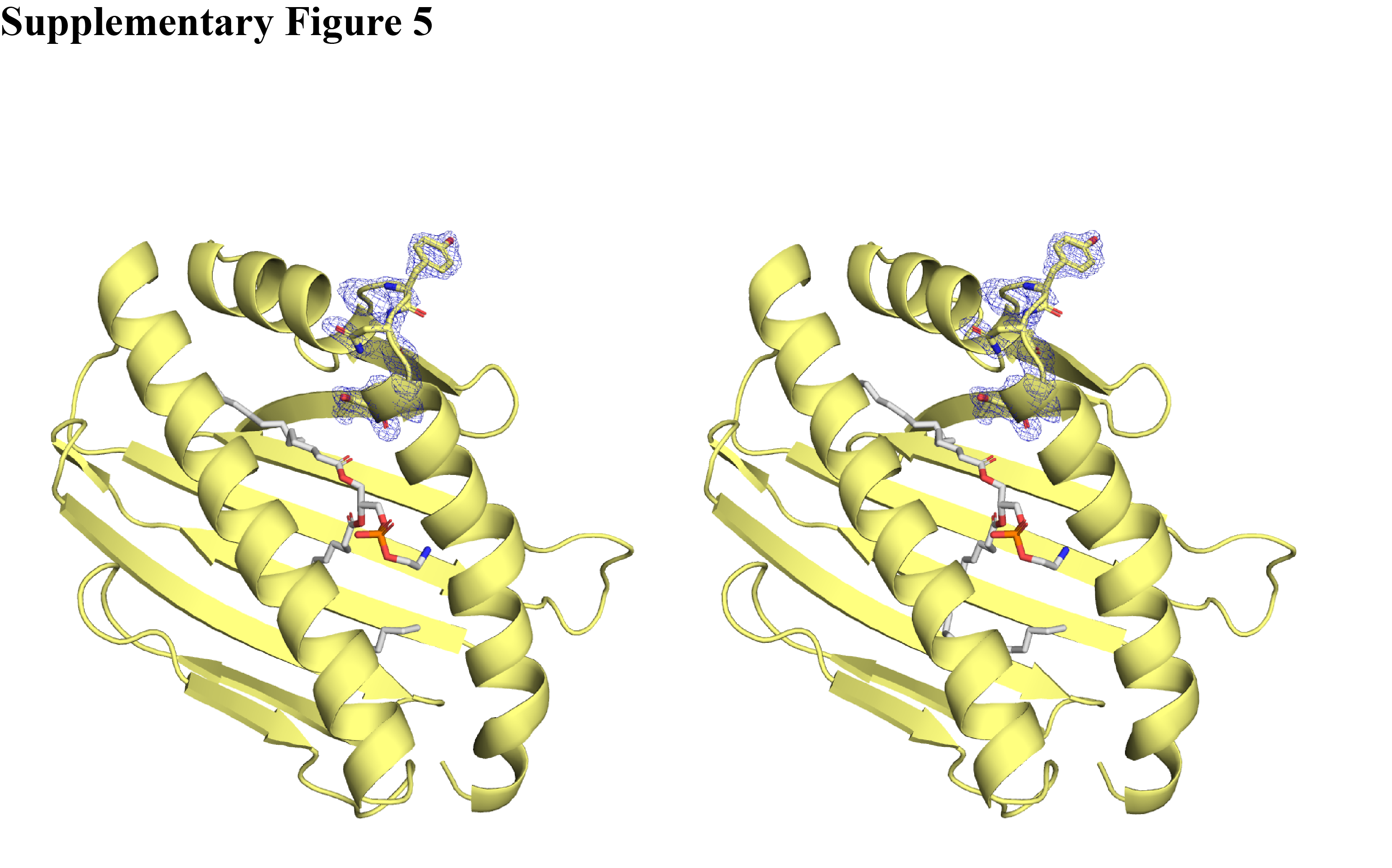

Supplement: Supplementary file 6 — Supplementary Figure 5. [file 41598_2024_53160_MOESM6_ESM.png]
